# Determining the predictors of preventive behaviors adopted by pregnant women against COVID-19 based on the Health Belief Model constructs: a cross sectional study

**DOI:** 10.1186/s12905-024-03305-7

**Published:** 2024-09-20

**Authors:** Roghieh Bayrami, Sima Masudi, Alireza Didarloo, Homeira Nournezhad

**Affiliations:** 1 Patient Safety Research Center, Clinical Research Institute, Midwifery Department, School of Nursing and Midwifery, Urmia University of Medical Sciences, Urmia, Iran; 2 Department of Biostatistics and Epidemiology, Urmia University of Medical Sciences, Urmia, Iran; 3 Social Determinants of Health Research Center, Clinical Research Institute, Department of Public Health, School of Public Health, Urmia University of Medical Sciences, Urmia, Iran; 4 Master degree in Consultation in midwifery, School of Nursing and Midwifery, Urmia University of Medical Sciences, Urmia, Iran

**Keywords:** COVID-19, Health Belief Model, Self-care, Pregnancy

## Abstract

**Background:**

Pregnant women face great challenges during the coronavirus disease 2019(COVID-19) pandemic. The purpose of this study was to explain the main dimensions of adoption of self-care behaviors against COVID-19 based on the health belief model(HBM) in pregnant women.

**Methods:**

This cross-sectional and analytical study was conducted in Iran, at the end of the third wave of the COVID-19 pandemic, between January and April 2021. Two hundred and thirty pregnant women who referred to Urmia health centers were selected using multi-stage random sampling. The data were collected using an online questionnaire including items that measured the participants’ demographic characteristics, the knowledge questionnaire, the HBM items, and questions assessing the adoption of self-care behaviors against COVID-19. The data were analyzed using SPSS software version 20. Descriptive statistics, bivariate Pearson’s correlation test, and multiple linear regression were used to analyze the data.

**Results:**

The results of this study showed that the rate of self-care behaviors against COVID-19 in the pregnant women participating in the present study was not very favorable. It was also shown that among the constructs of the HBM, knowledge, self-efficacy, and perceived barriers were the most important predictors of adopting self-care behaviors with a variance of 24% change among the pregnant women.

**Conclusion:**

Knowledge, self-efficacy, and perceived barriers were found in this study as the strongest predictors of self-care behaviors among pregnant women. Thus, it is suggested to implement interventions commensurate with the results of this study.

**Supplementary Information:**

The online version contains supplementary material available at 10.1186/s12905-024-03305-7.

## Introduction

Coronavirus disease 2019(COVID-19) is caused by a new type of coronavirus that was first identified in December 2019 in Wuhan, China. It has become a pandemic with far-reaching economic, social, and health consequences [1]. One of the most important aspects of Covid-19 is its rapid spread through airborne droplets and surfaces and objects contaminated with these droplets, which indicates the need for greater personal and social hygiene [2]. A large number of people in different countries were infected with COVID-19 due to rapid spread of the virus [3].

People with immune deficiencies who are at a greater risk of developing COVID-19 need a lot of care and attention from related health and care organizations. Suppression of relative immunity in pregnant women makes them more vulnerable to COVID-19. They experience immunological and physiological changes and may be more prone to viral respiratory infections [4]. Caring for these pregnant women can be complicated and difficult because the infection may adversely affect the mother, fetus, and baby [5]. Some researchers have reported the risk of preterm delivery, premature rupture of membranes, fetal tachycardia, and fetal distress during the third trimester of pregnancy among women infected with coronavirus [6].

Covid-19 pandemic poses several challenges for managing pregnant women [7]. Many government guidelines on self-care interventions have published. It includes social distancing, home quarantine, frequent hand washing with soap and water, mask-wearing, and hand rubbing with alcohol [8]. They also must be alert to any pregnancy related warning signs and inform their doctor immediately if they experience early symptoms such as fever, cough, fatigue, muscle aches, sore throat, and shortness of breath [9]. Online counselling by midwives and obstetricians for women can be useful to prevent the infection. Incorrect use of these vital public health precautions can lead to poor control and increased potential infection. Therefore, during this global pandemic, pregnant women demand a more considerate attitude and mutual understanding [10].

Planning and preparedness to deal with the COVID-19 crisis is one of the national and international necessities, and taking preventive measures at the community level to control the COVID-19 epidemics should be highly considered by policymakers and health officials [11].

There are a few theories and models that have been developed to identify health behaviors. The Health Belief Model (HBM) is a theoretical model that developed based on cognitive psychology and it can be used to predict health promotion and disease prevention behaviors. It also shows the relationship between health beliefs and health behaviors and emphasizes the intrinsic factors of individuals, such as knowledge, attitudes, beliefs, and behavior [12]. This model encompasses six main concepts, including: perceived benefits,  perceived susceptibility, perceived severity, perceived barriers, self-efficacy,  and cues to action [13]. According to the constructs of this model, to adopt preventive behaviors, people must first feel the danger (perceived susceptibility), then understand the depth and severity of the danger and the effects it may have on them and society. People should also believe in the effects and benefits of preventative measures and accept that taking preventive behaviors will cost less than getting the disease [13, 14].

The HBM has been used to assess many differing health behaviors among pregnant women in various contexts. The HBM has been shown to be a comprehensive framework regarding factors for improving eating behaviors and weight management [15, 16], and promoting physical activity [17, 18]. However, in various studies that have been conducted, different results of the impact of different structures of the health belief model on the adoption of health behaviors have been shown. For example, in some studies, the role of perceived susceptibility [19], or the effect of perceived benefits, perceived susceptibility, perceived barriers, and self-efficacy have been highlighted [20] and in other, from the HBM none of the constructs were significantly associated with COVID-19 vaccination [21].

Therefore, the aim of the present study was to examine the predictors of adopting preventive behaviors against COVID-19 based on the HBM constructs among the pregnant women who referred to the rural health centers in Urmia city, Iran. In the field of health education, by examining the impact of health belief model structures in adopting preventive behavior, it is possible to find suitable strategies for promoting self-care behaviors in pregnant women. These can be key indicators for practitioners and researchers alike in promoting health outcomes for pregnant women.

## Materials and methodology

### Study type and participants

This descriptive-analytical cross-sectional study was performed on 230 pregnant women who referred to Urmia health centers at the end of the third wave of the COVID-19 pandemic, between January and April 2021.

### Sample size

The sample size was estimated using the following equation [22]:

$$n \ge 1 + \frac{{p\left( {1 - R_{adj}^2} \right)}}{\delta }$$

Where, *p* is the number of variables in the regression model, *R*_*2adj*_ is the adjustment coefficient, and *δ* is the difference between the coefficient of determination and the adjusted coefficient of determination. Due to the lack of a similar study in this field, preventive measures against the Zika virus in Thailand were used to estimate the value of the adapted coefficient of determination, and δ showing the difference between the coefficient of determination and the coefficient of determination was estimated from a previous study by Siramanrate [23]. Thus, using the above equation (*p* = 11; R2 = 0.307; δ = 0.048), the required sample size was estimated as159 people. Considering about 30% non-response rate, the sample size was increased to 230 people.

### Eligibility and recruitment

The study inclusion criteria consisted of (a) any adult pregnant woman (18–49 years old) living in Urmia, (b) having reading and writing skills in Persian, (c) an individual with access to a smart cell phone and WhatsApp, and (c) those who provided consent to participate in the study. Incomplete questionnaires were considered as the exclusion criteria. Based on the socioeconomic and cultural conditions of the regions hosting, the health centers were classified into three categories: high, moderate, and low level, including 26, 20, and 20 centers, respectively. Four centers from each category were selected randomly. Based on the centers’ population coverage, the sample size for each center determined. In each center, a list of pregnant women who were literate and free of mental illness (eligibility criteria) was prepared. The participants of the study selected using simple random sampling from the list of each center. Then the landline number and/or mobile phone number was extracted from the information system of the center. The researcher called the pregnant women and explained the study and its objectives. Those women who agreed to participate received a link for filling in an online questionnaire.

### Data collection tools

The data were gathered using a researcher–made questionnaire consisted of four sections. (1) the demographic and midwifery characteristics, (2) the knowledge questionnaire, (3) the questionnaire based on constructs of the HBM and (4) the self-care behavior questionnaire. The demographic and midwifery questionnaire contains personal information such as age, education, occupation, family income, number of pregnancies and live children. The second part of the questionnaire contained 22 items with true-false answer options that assessed the participants’ awareness towards COVID-19 (e.g. Can COVID-19 be asymptomatic?). One mark was given for a correct answer and zero for an incorrect or no answer. The total score for each respondent varied from 0 to 22.

The third section of the questionnaire includes 43 questions, which assess the six constructs of the health belief model to perform self-care behaviors to prevent COVID-19 as follows: 1- Perceived susceptibility(7 questions, score range: 7 to 35): “If I do not wear a mask outdoors, I may become infected with the Coronavirus”, 2- Perceived severity (7 questions, score range: 7 to 35): “Mortality rate due to covid-19 is high”, 3- Perceived benefits (7 questions, score range: 7 to 35): “Washing my hands regularly prevents me from becoming infected with the coronavirus”,4- Perceived barriers (6 questions, score range: 6 to 30): “It is difficult for me to provide the face mask “, 5- Self-efficacy (6 questions, score range: 6 to 30): “I am confident in my ability to disinfect my environment”, and 6- Cues to action (10 questions, score range: 0 to 10):“Have you read about the importance and necessity of disinfecting food and the environment through virtual space?” Except cues to action, the questions of all constructs were on a five-point Likert scale (scoring from 1= ‘strongly disagree’ to 5= ‘strongly agree’). The items on the Cues to action had a yes/no answer that scored as zero/one.

The fourth section (self-care behavior) consisted of 10 items (score range: 0 to 40): “I observe a distance of at least 1.5 meters in dealing with others”. The items were scored on a 4-point Likert scale (ranging from 0= ‘Never’ to 4= ‘Always’).

The researcher made questionnaires were developed by the researchers. In order to check the validity of self-made questionnaires in this study, face validity, content validity index (CVI) and content validity ratio (CVR) were conducted using both qualitative and quantitative methods. The opinions of 10 specialists including five reproductive health experts, three health education and promotion experts, and two epidemiologists were assessed. The acceptable CVR rate according to the number of experts in the present study was 0.62 and above. The CVI and CVR for all the dimensions of the questionnaire were higher than 0.80 and 0.67, respectively. The internal consistency of the questionnaire was confirmed by Cronbach’s alphas, which were 0.87, 0.7, 0.76, 0.71, 0.78, 0.80, and 0.92 for perceived susceptibility, perceived severity, perceived benefits, perceived barriers, self-efficacy, cues to action, and self-care behaviors, respectively.

### Data analysis

Data analysis was performed in SPSS (version 20) using Pearson’s correlation coefficient and linear regression analysis. *P*-values less than 0.05 were considered statistically significant.

This study was approved by the local Ethics Committee of Urmia University of Medical Sciences, Urmia, Iran (IR.UMSU.REC.1399.142). Information about confidential data management and voluntary withdrawal from the study was provided to participants and informed written consent was obtained from each of them.

## Results

Two hundred thirty pregnant women with a mean age of 29.57 ± 5.82 participated in this study. Of whom214 were housewives, and 16 were employed. Table 1 summarizes the participants’ demographic and midwifery characteristics.

**Table 1:** Demographic and midwifery characteristics of pregnant women who participated in the study(*n* = 230)

Demographic Characteristics	Number (Percentage of The Sample)
Employment status	
Housewife	214(93.0)
Employed	16(7.0)
Education level	
Less than high school	54(23.4)
High school diploma	143(62.2)
Bachelor’s degree and higher	33(14.3)
Income Adequacy	
Inadequate Relatively adequate Adequate	60(26.08) 128(55.65) 42(18.26)
Having health insurance	
Yes	222(96.5)
No	8(3.5)
Pregnancy number	
1	68(29.6)
2	94(40.9)
3	52(22.6)
4+	16(6.9)
Children number	
0	74(32.2)
1	106(46.1)
2	42(18.3)
3	8(3.5)
COVID-19 history	
Yes	23(10.0)
No	207(90.0)

The mean score of participants’ knowledge was 17.04 ± 1.90. The minimum level of knowledge was 11 and the maximum level of knowledge was 21. The mean perceived severity score was 21.57 ± 3.92, perceived benefits were 28.86 ± 3.38, perceived barriers were 25.29 ± 3.53, perceived susceptibility was 30.12  ± 3.44, perceived self efficacy was 21.50 ± 2.72, and cues to action was 8.09 ± 2.24. The mean score of self-care behaviors associated with COVID-19 (17.27 ± 5.44) was not very favorable (Table 2).

**Table 2:** Mean scores of knowledge, health belief model constructs and self-care behaviors regarding prevention of COVID-19

Variable	Mean ± SD	Minimum	Maximum
Knowledge	17.04 ± 1.90	11	21
Perceived susceptibility	30.12 ± 3.44	22	35
Perceived severity	21.57 ± 3.92	14	30
Perceived benefits	28.86 ± 3.38	23	35
Perceived barriers	25.29 ± 3.53	19	33
Self-efficacy	21.50 ± 2.72	17	29
Cues to action	8.09 ± 2.24	3	10
Self-care behavior	17.27 ± 5.44	5	27

Except for perceived benefits, we found significant correlations between the constructs of the health belief model and self-care behaviors related to the prevention of COVID-19. The correlations were negative for perceived severity, and perceived barriers (Table 3).

**Table 3:** Pearson correlation coefficient between individual factors and structures of the health belief model in relation to self-care behaviors regarding prevention of COVID-19

		knowledge	perceived susceptibility	perceived severity	perceived benefits	perceived barriers *t*	self-efficacy	cues to action
Self-care behavior	r	0.346	0.147	-0.275	0.019-	-0.247	0.253	0.264
	*P*	< 0.001	0.026	< 0.001	0.777	< 0.001	< 0.001	< 0.001

We used a multiple regression model using backward method to predict self-care behaviors of pregnant women against to COVID-19 from the structures of the health belief model, knowledge, age, and number of children. From the variables entered in the model, perceived barriers, self-efficacy, and knowledge statistically significantly predicted self-care behaviors, F (5, 225) = 17.296, *p* < 0.001, R^2^ = 0.235 (Table 4).

**Table 4:** Multivariate linear regression model in predicting self-care behavior of pregnant women against COVD-19

HBM^*^ constructs	B	Std. Error	Standardized Beta	sig	95%CI for B	
					lower	upper
Constant	7.536	5.760		0.259	-4.335	16.028
Perceived severity	-0.184	0.098	-0.133	0.060	-0.414	-0.042
Perceived barrier	-0.239	0.095	-0.155	0.013	-0.638	-0.211
Self-efficacy	0.381	0.124	0.191	0.002	0.421	0.941
Knowledge	0.678	0.201	0.236	0.001	0.425	1.214

^*^Health Belief Model; Model summary: *R* = 0.485, R square = 0.235, Adjusted R square = 0.222

## Discussion

This study aimed to investigate the predictive factors associated with the adoption of self-care behaviors for preventing COVID-19 disease in pregnant women using the HBM. The pregnant women had a good knowledge about COVID-19 but their self-care behaviors were not favorable. It was also shown that among the constructs of the HBM, knowledge, self-efficacy, and perceived barriers were the most important predictors of adopting self-care behaviors with a variance of 24% change among the pregnant women.

Similarly, Khazaei Poor [24] reported that the constructs of the health belief model could explain 26% of self-care behaviors effective in the prevention of COVID-19.

The results of this study showed that the average score of adopting self-care behaviors in preventing COVID-19 was about 17.27, showing that self-care behaviors taken by the pregnant women to prevent COVID-19 disease were not desirable due to the high prevalence of the disease in Iran. However, some studies [25–27] estimated preventive function of different study groups as desirable. Given the specific physiological conditions of pregnant women, they need to pay attention to the plans and strategies adopted to care for COVID-19 disease. Considering the possible beneficial effects of adopting self-care behaviors in health-promoting and reducing the risk of the COVID-19 epidemic, it is necessary to adopt an appropriate strategy to remove possible obstacles to managing this global epidemic.

We found statistically significant correlations between self-care behaviors with knowledge and constructs of HBM except for perceived benefits. However, this correlation was reversed for perceived severity and perceived barriers, indicating that those with high scores in these constructs are less likely to engage in self-care behaviors to prevent COVID-19. This finding is consistent with the results of previous studies [3, 28].

In this study, knowledge of pregnant women about the COVID-19 and its transmission ways was high, and it was the first determinant factor in adopting self-care behaviors. During the pandemic period, people received a lot of information about the disease nature and its effects on the health and lives in various ways, such as mass media, virtual networks, and messages published by the Ministry of Health. With increasing awareness, people are more likely to do optimal self-care behaviors to prevent COVID-19 disease [24].

Perceived self-efficacy had a positive and direct effect on preventive behaviors against COVID-19. The more people feel motivated, capable, and hoping to succeed in fighting the coronavirus, they show more willingness to do individual health behaviors [29]. In the present study, self-efficacy was conceptualized to the extent to which a person feels that they can use self-care and preventive strategies to combat the coronavirus.

Perceived barriers construct as the third construct of the HBM was able to account for the adoption of self-care behavior. In the present study, limited access to essential personal protective equipment was one of the barriers to women’s self-care behaviors. In a previous study perceived barriers was the most important construct of the HBM in predicting the behavior [30]. It should be noted that the low perceived barriers are a privilege because the individuals believe that they face fewer obstacles and fewer problems in adopting self-care and preventive behaviors. In line with the findings of this study, several studies reported the Iranian health care facilities have faced equipment and supplies management challenges in managing COVID-19 pandemic [31, 32].

Besides, the objective and psychological costs of the recommended activities are low, or they are preferable due to the benefits of the behavior. Therefore, it can be suggested that by performing a series of interventions and adopting effective policies to reduce barriers as much as possible, the possibility of adopting self-care behaviors for preventing COVID-19 increases.

Perceived severity as the fourth construct of the HBM could predict the adoption of self-care behaviors in confronting COVID-19. Perceived severity is ultimately related to the perceived threat and refers to the extent to which individuals perceive the danger and seriousness of the spread of coronavirus. This finding is consistent with the results of previous studies by Bates et al. [33], and Khazaee-Pool et al. [24]

Although the present study showed a positive and significant correlation between perceived susceptibility, perceived benefits, and cues to action in the adoption of self-care, these constructs in the model extracted from regression analysis were not confirmed as predictive constructs. In fact, if people are aware of the benefits of taking preventative behaviors, they are better prepared to take activities and they are more likely accept the mentioned behaviors. In other words, if a person believes that home quarantine and the use of personal protective equipment can reduce the risk of developing the disease or transmitting it to others, or have social benefits such as reducing treatment costs or potential costs to the health system of the country, they will be more likely to do self-care behaviors [34, 35].

In tong et al. study cues-to-action was found to be positively associated with adherence to COVID-19 precautionary measures [36]. This finding is consistent with our results. Cues to action can act as stimulus received by people from the COVID-19 outbreak. This stimulus can be in the form of a clip, news of the death of people due to COVID-19, the effects and consequences of this disease on people’s lives posted on mass media and social networks, or the advice and warnings of the staff of health centers [35, 37]. Most of the women in this study reported that the most important sources of information about COVID-19 were health workers and warnings from the Ministry of Health through the mass media. Given the importance of mass media, especially in the period of growth of new technologies and virtual networks and given the importance of non-aggregation to reduce the transfer of COVID-19, the high potentials of social media can be used for educational, awareness-raising, and behavior change purposes.

Of course, it should be noted that pregnant women’s engagement in self-care can be affected by some non-behavioral environmental factors such as the availability of facilities and preventive devices, high cost of protective equipment and disinfectants, and strategies adopted nationwide, etc. By informing the community and officials and adopting useful policies and effective interventions, it is possible to increase the adoption of self-care and preventive behaviors against COVID-19.

This study has a strong point considering that it has been demonstrated that determinants associated with self-care behaviors during COVID-19 pandemic among pregnant women. It can be used to develop future interventions that target these psychological factors related to preventive behaviors during pregnancy. The use of online surveys was considered for sampling so there is a chance of bias in selection.

Based on this, it is suggested that future interventional studies be conducted on self-care behaviors related to coronavirus. It is suggested that other studies in the field of self-care be performed with other behavioral models.

## Conclusion

Knowledge, self-efficacy, and perceived barriers were found in this study as the strongest predictors of self-care behaviors among pregnant women. The results of this study can be used by managers at local, and national levels in planning interventions commensurate with the results of this study.

## Electronic Supplementary Material

Below is the link to the electronic supplementary material.

Supplementary Material 1

## Data Availability

The data used and/or analyzed during the current study are available from the corresponding author on reasonable request.

## References

[1] Mahase E. China coronavirus: WHO declares international emergency as death toll exceeds 200. BMJ: Br Med J (Online). 2020;368. 10.1136/bmj.m408 32005727

[2] Zhai P, Ding Y, Wu X, Long J, Zhong Y, Li Y. The epidemiology, diagnosis and treatment of COVID-19. Int J Antimicrob Agents. 2020;55(5):105955. 32234468 10.1016/j.ijantimicag.2020.105955 PMC7138178

[3] Doshmangir L, Mahbub Ahari A, Qolipour K, Azami-Aghdash S, Kalankesh L, Doshmangir P, et al. East Asia’s strategies for effective response to COVID-19: lessons learned for Iran. Manage Strategies Health Syst. 2020;4(4):370–3.

[4] Liu H, Wang LL, Zhao SJ, Kwak-Kim J, Mor G, Liao AH. Why are pregnant women susceptible to COVID-19? An immunological viewpoint. J Reprod Immunol. 2020;139:1–5. 10.1016/j.jri.2020.103122 PMC7156163 32244166

[5] Sahu KK, Lal A, Mishra AK. COVID-2019 and pregnancy: a plea for transparent reporting of all cases. Acta Obstet Gynecol Scand. 2020;99(7). 10.1111/aogs.13850 PMC7228243 32191350

[6] Khan S, Peng L, Siddique R, Nabi G, Xue M, Liu J, et al. Impact of COVID-19 infection on pregnancy outcomes and the risk of maternal-to-neonatal intrapartum transmission of COVID-19 during natural birth. Infect Control Hosp Epidemiol. 2020;41(6):748–50. 32279693 10.1017/ice.2020.84 PMC7156579

[7] Maharlouei N, Asadi N, Bazrafshan K, Roozmeh S, Rezaianzadeh A, ZahedRoozegar, et al. Knowledge and attitude regarding COVID-19 among pregnant womenin southwestern Iran in the early period of its outbreak: a cross-sectional study. Am J Trop Med Hyg. 2020;103:2368–75. 33124530 10.4269/ajtmh.20-0608 PMC7695057

[8] Chiu NC, Chi H, Tai YL, Peng CC, Tseng CY, Chen CC, et al. Impact of wearing masks, Hand Hygiene, and Social Distancing on Influenza, Enterovirus, and all-cause Pneumonia during the Coronavirus Pandemic: Retrospective National Epidemiological Surveillance Study. J Med Internet Res. 2020;22(8):e21257. 32750008 10.2196/21257 PMC7471891

[9] Liang H, Acharya G. Novel corona virus disease (COVID-19) in pregnancy: what clinical recommendations to follow? Acta Obstet Gynecol Scand. 2020;99(4):439–42. 32141062 10.1111/aogs.13836

[10] Besho M, Tsegaye R, Yilma MT, Kasaye HK, Tolossa T, Hiko N, et al. Knowledge, attitude and practice toward Corona virus infection among pregnant women attending Antenatal Care at Public hospitals in three wollega zones, Ethiopia. Int J Gen Med. 2021;14:3563–73. 34290526 10.2147/IJGM.S295490 PMC8289464

[11] Wu Z, McGoogan JM. Characteristics of and important lessons from the coronavirus disease 2019 (COVID-19) outbreak in China: summary of a report of 72 314 cases from the Chinese Center for Disease Control and Prevention. JAMA. 2020;323(13):1239–42. 32091533 10.1001/jama.2020.2648

[12] Bakhtiar K, Bastami F, Sharafkhani N, Almasian M. The psychological determinants of self-medication among the elderly: an explanation based on the Health Belief Model. Elder Health J. 2017;3(2):59–66.

[13] Glanz K, Rimer BK, Viswanath K. Health behavior and health education: theory, research, and practice. Wiley; 2008.

[14] Sharafkhani N. Survey of prostate cancer-preventive behaviors based on the health belief model constructs among male teachers of Urmia city, in 2015. Nurs Midwifery J. 2016;14(3):271–81.

[15] Mohebi S, Sharifirad GH, Rasekhi H, Matlabi M, Shahsiah M, Tabbaraie Y. Effect of nutrition education program on the recommended weight gain during pregnancy—application of Health Belief Model: a randomized clinical trial. Qom Universityof Med Sci J. 2012;6:23–30.

[16] Abdolaliyan N, Hossein S, Kzemi A, Hasanzadeh A. Determinants of the self-efficacy of physical activity for maintaining weight during pregnancy: the application of the Health Belief Model. J Educ Health Promotion. 2017;6:93. 10.4103/jehp.jehp_175_16 PMC5651666 29114560

[17] Kazemi A, Shafieian M. A randomized trial to promote physical activity during pregnancy based on health belief model. J Educ Health Promotion. 2017;6:40. 10.4103/jehp.jehp_19_15 PMC5441198 28584839

[18] Yu H, He J, Szumilewicz A. Pregnancy activity levels and impediments in the era of COVID-19 based on the Health Belief Model: a cross-sectional study. Int J Environ Res Public Health. 2022;19:3283. 35328974 10.3390/ijerph19063283 PMC8954454

[19] Hatamzadeh N, Shakerinejad G, Navak T, Haghi M, Haghighizadeh MH, Baumann SL. The efficiency of the Health Belief Model in Predicting the preventive behaviors of pregnant women during the COVID-19 pandemic in Iran. Nurs Sci Q. 2024;37(1):76–80. 38054312 10.1177/08943184231207384

[20] Karimy M, Bastami F, Sharifat R, Heydarabadi AB, Hatamzadeh N, Pakpour AH, et al. Factors related to preventive COVID-19 behaviors using health belief model among general population: a cross-sectional study in Iran. BMC Public Health. 2021;21(1):1934. 34689728 10.1186/s12889-021-11983-3 PMC8542411

[21] Ayieko S, Markham C, Baker K, Messiah SE. Psychological determinants of COVID-19 vaccination uptake among pregnant women in Kenya: a Comprehensive Model Integrating Health Belief Model constructs, anticipated regret, and trust in Health authorities. COVID. 2024;4;749–760.

[22] Riley RD, Snell KI, Ensor J, Burke DL, Harrell FE Jr, Moons KG, et al. Minimum sample size for developing a multivariable prediction model: part I–Continuous outcomes. Stat Med. 2019;38(7):1262–75. 30347470 10.1002/sim.7993

[23] Siramaneerat I. Perception of the Zika virus infection and its influence on Zika prevention practices by pregnant women at the region 5 Health Promotion Center in Thailand. Med J Indonesia. 2018;27(3):201–8.

[24] Khazaee-Pool M, Shahrvsand S, Naghibi SA. Predicting Covid-19 preventive behaviors based on Health Belief Model: an internet-based study in Mazandaran Province, Iran. J Mazandaran Univ Med Sci. 2020;30(190):56–66.

[25] Fallahi A, Mahdavifar N, Ghorbani A, Mehrdadian P, Mehri A, Joveini H, et al. Public knowledge, attitude and practice regarding Home Quarantine to prevent COVID-19 in Sabzevar city, Iran. J Military Med. 2020;22(6):580–8.

[26] Kwok K, Li K, Chan H, Yi Y, Tang A, Wei W. Community responses during early phase of COVID-19 epidemic, Hong Kong [published online ahead of print, 2020 Apr 16]. Emerg Infect Dis. 2020;26(7):103201. 10.3201/eid2607.200500 PMC7323558 32298227

[27] Lin Y, Hu Z, Alias H, Wong LP. Influence of mass and social media on psychobehavioral responses among medical students during the downward trend of COVID-19 in Fujian, China: cross-sectional study. J Med Internet Res. 2020;22(7):e19982. 32584779 10.2196/19982 PMC7373377

[28] Delshad Noghabi A, Yoshany N, Mohammadzadeh F, Javanbakht S. Predictors of Covid-19 preventive behaviors in Iranian Population over 15 Years Old: an application of Health Belief Model. J Mazandaran Univ Med Sci. 2020;30(191):13–21.

[29] Groenewold G, de Bruijn B, Bilsborrow R. Psychosocial factors of migration: adaptation and application of the health belief model. Int Migration. 2012;50(6):211–31.

[30] Weinstein ND. Perceived probability, perceived severity, and health-protective behavior. Health Psychol. 2000;19(1):65. 10711589 10.1037//0278-6133.19.1.65

[31] Tahernejad S, Ghaffari S, Ariza-Montes A, Wesemann U, Farahmandnia H, Sahebi A. Post-traumatic stress disorder in medical workers involved in earthquake response: a systematic review and meta-analysis. Heliyon. 2023;9(1):e12794. 36685451 10.1016/j.heliyon.2023.e12794 PMC9850193

[32] Abbari A, Salahi S, Hadian M, khakdel Z, Hosseini E, Sheikhbardsiri H. Exploring the challenges of Iranian government hospitals related to Covid-19 pandemic management: a qualitative content analysis research from the nurses perspective. BMC Nursing.2022; 21(226). 10.1186/s12912-022-01008-8 PMC9372986 35962433

[33] Bates BR, Villacís AG, Mendez-Trivino A, Mendoza LE, Grijalva MJ. Determinants of intentions to prevent triatomine infestation based on the health belief model: an application in rural southern Ecuador. PLoS Negl Trop Dis. 2020;14(1):e0007987. 31999721 10.1371/journal.pntd.0007987 PMC6991950

[34] Huang X, Dai S, Xu H. Predicting tourists’ health risk preventative behaviour and travelling satisfaction in Tibet: combining the theory of planned behaviour and health belief model. Tourism Manage Perspect. 2020;33:100589.

[35] Akter J. Human Behavior toward COVID-19. Available on the https://www.researchgate.net/publication/340066427_Title_Human_Behavior_toward_COVID-19. 2020.

[36] Tong KK, Chen JH, Yu EW, Wu AMS. Adherence to COVID-19 Precautionary Measures: Applying the Health Belief Model and Generalised Social Beliefs to a Probability Community Sample. Applied Psychology: Health and Well-Being. 2020;12(4):1205–1223. 10.1111/aphw.12230 PMC7675419 33010119

[37] Ayosanmi OS, Oden L, Ayosanmi T, Alli B, Wen M, Johnson J. The role of health belief model in HIV screening decision among international students in the United States: a pilot study. Int J Translational Med Res Public Health. 2020;4(1):4–12.

