# Supplementary material for: Determining the predictors of preventive behaviors adopted by pregnant women against COVID-19 based on the Health Belief Model constructs: a cross sectional study
Source: BMC Womens Health. 2024 Sep 20;24:528. doi: 10.1186/s12905-024-03305-7 (PMC11414123; doi:10.1186/s12905-024-03305-7)
Supplement: Supplementary file 1 — Supplementary Material 1 [file 12905_2024_3305_MOESM1_ESM.docx]

Questionnaires

**Awareness questions**

| **Items** | **Yes** | **No** |
| --- | --- | --- |
| 1. Is COVID-19 common? |  |  |
| 2. Does the corona virus cause acute respiratory problems? |  |  |
| 3. Can the corona virus be transmitted through contaminated hands? |  |  |
| 4. Is the corona virus transmitted through kissing? |  |  |
| 5. Is the corona virus transmitted through sneezing and coughing? |  |  |
| 6. Is the corona virus transmitted through contact with body fluids or blood? |  |  |
| 7. Is the corona virus transmitted through vaginal sexual contact? |  |  |
| 8. Can the corona virus be transmitted through the shared use of personal equipment? |  |  |
| 9. Can COVID-19 be asymptomatic? |  |  |
| 10. Is there vaccine for COVID-19? |  |  |
| 11. Can corona disease be treated? |  |  |
| 12. Can COVID-19 be treated with antibiotics? |  |  |
| 13. Is the corona virus transmitted from mother to fetus? |  |  |
| 14. Does the mother's infection with the corona virus cause the death of the fetus? |  |  |
| 15. Does mother's infection with corona virus cause premature delivery? |  |  |
| 16. Does the mother's infection with the corona virus cause fetal abnormalities? |  |  |
| 17. Is the corona virus transmitted through breast milk? |  |  |
| 18. Does wearing gloves prevent virus transmission? |  |  |
| 19. Does wearing a mask prevent the transmission of the virus? |  |  |
| 20. Is alcohol the main disinfectant to prevent virus transmission? |  |  |
| 21. Does staying at home prevent corona virus transmission? |  |  |
| 22. Dose the COVID-19 vaccine stop someone from getting the virus? |  |  |

**Questions based on the HBM constructs**

| **Perceived Susceptibility** | Strongly agree | Agree | Neither agree nor disagree | Disagree | Strongly disagree |
| --- | --- | --- | --- | --- | --- |
| 1. If I do not wear a mask outdoors, I may become infected with the Coronavirus. |  |  |  |  |  |
| 2.If I do not wear gloves outdoors, I may become infected with the Coronavirus. |  |  |  |  |  |
| 3. If I touch contaminated surfaces, I may become infected with the Coronavirus. |  |  |  |  |  |
| 4. If I don't disinfect the fruits and vegetables, I may become infected with the Coronavirus. |  |  |  |  |  |
| 5. My baby is at risk for COVID‑19. |  |  |  |  |  |
| 6. If I do not wash my hands regularly, I may become infected with the Coronavirus. |  |  |  |  |  |
| 7. If I do not keep at least 1.5 meters away from people, I may become infected with the Coronavirus. |  |  |  |  |  |

| **Perceived Severity** | Strongly agree | Agree | Neither agree nor disagree | Disagree | Strongly disagree |
| --- | --- | --- | --- | --- | --- |
| 1. Mortality rate due to covid-19 is high. |  |  |  |  |  |
| 2. If I get infected with the corona virus, I will die. |  |  |  |  |  |
| 3. If I get infected with corona virus, I will get severe lung disease. |  |  |  |  |  |
| 4. If I get infected with corona virus, I will get severe gastrointestinal disease. |  |  |  |  |  |
| 5. If I get infected with the corona virus, the fetus inside the womb will also get infected. |  |  |  |  |  |
| 6. If I get infected with the corona virus, the fetus inside the womb will also die. |  |  |  |  |  |
| 7. If I get infected with corona virus, the treatment will be difficult and long-term. |  |  |  |  |  |

| **Perceived Benefits** | Strongly agree | Agree | Neither agree nor disagree | Disagree | Strongly disagree |
| --- | --- | --- | --- | --- | --- |
| 1. Wearing glove prevents me/my baby from becoming infected with the coronavirus |  |  |  |  |  |
| 2. Wearing a mask prevents me/my baby from becoming infected with the coronavirus |  |  |  |  |  |
| 3. Washing my hands regularly prevents me/my baby from becoming infected with the coronavirus |  |  |  |  |  |
| 4. Disinfecting my surroundings prevents me/my baby from becoming infected with the coronavirus. |  |  |  |  |  |
| 5. Washing things with soap and water after shopping prevents me/my baby from becoming infected with the coronavirus. |  |  |  |  |  |
| 6. Staying at home prevents me/my baby from becoming infected with the coronavirus. |  |  |  |  |  |
| 7. keeping a physical distance from others (at least 1.5 meters) prevents me/my baby from becoming infected with the coronavirus. |  |  |  |  |  |

| **Perceived Barriers** | Strongly agree | Agree | Neither agree nor disagree | Disagree | Strongly disagree |
| --- | --- | --- | --- | --- | --- |
| 1. It is difficult for me to provide the face mask. |  |  |  |  |  |
| 2. It is difficult for me to provide the glove. |  |  |  |  |  |
| 3. It is difficult for me to provide the disinfectant (alcohol). |  |  |  |  |  |
| 4. It is difficult for me to breathe with face mask. |  |  |  |  |  |
| 5. I am afraid of complications of alcohol. |  |  |  |  |  |
| 6. I am afraid of whitex solution complications. |  |  |  |  |  |

| **Perceived Self-efficacy** | Strongly agree | Agree | Neither agree nor disagree | Disagree | Strongly disagree |
| --- | --- | --- | --- | --- | --- |
| 1. I am confident in my ability to keep at least 1.5 meters away from people. |  |  |  |  |  |
| 2 I am confident in my ability to wear a mask even if it is expensive. |  |  |  |  |  |
| 3. I am confident in my ability to wear glove even if they are expensive. |  |  |  |  |  |
| 4. I am confident in my ability to stay at home even if I am bored. |  |  |  |  |  |
| 5. I am confident in my ability to wash my hands regularly. |  |  |  |  |  |
| 6.I am confident in my ability to disinfect my environment. |  |  |  |  |  |

| **Cues to Action** | **Yes** | **No** |
| --- | --- | --- |
| 1. Have you heard about the necessity of staying at home, washing hands/wearing masks and gloves through TV and radio? |  |  |
| 2. Did the doctor/health workers give advice or guidance to stay at home/wash hands/wear masks and gloves? |  |  |
| 3. Have you read an article about the importance and necessity of washing hands/wearing masks and gloves, and staying at home through cyberspace? |  |  |
| 4. Have you heard anything from your friends and family about the importance of staying at home, washing your hands/wearing masks and gloves? |  |  |
| 5. Have you heard anything about the need to disinfect food and the surrounding environment through TV and radio? |  |  |
| 6. Did the doctor/health care workers have advice or guidance for disinfecting food and the surrounding environment? |  |  |
| 7. Have you read an article about the importance and necessity of disinfecting food and the surrounding environment through virtual space? |  |  |
| 8. Have you heard from your friends and family about the importance of disinfecting food and the surrounding environment? |  |  |
| 9. Have you ever read a pamphlet or book about COVID-19? |  |  |
| 10. Have you ever seen a poster related to COVID-19? |  |  |

**Preventive Behaviors**

| **Items of preventive behaviors** | Always | Often | Sometimes | Never |
| --- | --- | --- | --- | --- |
| 1. I stay at home and I don't go out except for essential cases. |  |  |  |  |
| 2.I observe a distance of at least 1.5 meters in dealing with others |  |  |  |  |
| 3. I wear gloves outside the house. |  |  |  |  |
| 4. I wear a mask outside the house. |  |  |  |  |
| 5. I regularly wash my hands with soap and water for at least 30 seconds. |  |  |  |  |
| 6. I disinfect fruits and vegetables. |  |  |  |  |
| 7. I clean contaminated surfaces with disinfectants. |  |  |  |  |
| 8. I put the outer clothes in a place out of reach. |  |  |  |  |
| 9. At the time of buying the necessary things, I immediately wash them with soap and water. |  |  |  |  |
| 10.  I avoid shaking hands with others or kissing others. |  |  |  |  |
